# *In vitro* susceptibility profiles of 16 antifungal drugs against *Trichophyton indotineae*

**DOI:** 10.1128/spectrum.00618-25

**Published:** 2025-06-23

**Authors:** Wenting Xie, Xue Kong, Hailin Zheng, Huan Mei, Naicen Ge, Weida Liu, Guanzhao Liang, Xiaofang Li

**Affiliations:** 1 Department of Medical Mycology, Hospital for Skin Diseases, Institute of Dermatology, Chinese Academy of Medical Sciences & Peking Union Medical College, Nanjing, China; 2 Jiangsu Key Laboratory of Molecular Biology for Skin Diseases and STIs, Nanjing, China; 3 CAMS Collection Centre of Pathogen Microorganisms-D (CAMS-CCPM-D), Nanjing, China; University of Texas Medical Branch at Galveston, Galveston, Texas, USA

**Keywords:** *Trichophyton indotineae*, antifungal susceptibility, azoles, echinocandins, amphotericin B

## Abstract

The worldwide emergence of *Trichophyton indotineae* has garnered significant attention due to its growing resistance to conventional antifungal therapies, posing significant challenges to the effective management of dermatophytosis. The *in vitro* activities of 16 antifungals were tested against 37 *T. indotineae* isolates using the broth microdilution method according to Clinical and Laboratory Standards Institute protocol M38-A3. Luliconazole and amorolfine exhibited low MICs, while itraconazole showed moderately decreased susceptibility, making them preferred treatment options. Echinocandins demonstrated favourable *in vitro* activity against *T. indotineae*, but their potential as therapeutic alternatives for recalcitrant dermatophytosis remains uncertain due to pharmacokinetic and pharmacodynamic limitations. In contrast, terbinafine, fluconazole, griseofulvin, oteseconazole, isavuconazole, ravuconazole, and amphotericin B exhibited elevated MICs for many strains, with strong correlations between certain azoles, indicating potential cross-resistance. A difference in the susceptibility to terbinafine and voriconazole between Chinese and Indian isolates was observed. These findings could provide valuable insights into antifungal resistance patterns and help guide treatment strategies for *T. indotineae* infections.

**IMPORTANCE:**

*Trichophyton indotineae* is a novel emerging dermatophyte that has rapidly spread globally in recent years. Its resistance to first-line antifungal agents, particularly terbinafine and fluconazole, has significantly limited treatment options, emphasizing the urgent need for alternative therapies. This study provides comprehensive antifungal susceptibility data for 16 available antifungal agents, offering valuable insights into potential treatment strategies.

## OBSERVATION

The rise of resistance to the conventional antifungals *Trichophyton indotineae* has posed a significant challenge to dermatophytosis treatment. Although initially reported from India (1), *T. indotineae* has rapidly emerged in nearly 40 countries around the world over the past 5 years and has become a significant global public health concern (2, 3). Epidemiological surveys from India (4), Canada (5), and the USA (6) have shown that more than 70% of *T. indotineae* isolates are resistant to terbinafine, the first-line drug for dermatophytosis (7). Additionally, griseofulvin and triazoles, including fluconazole, itraconazole, and voriconazole, have been reported to have elevated minimum inhibitory concentrations (MICs) against some isolates (4, 8). In 2022–2023, we identified 13 cases of refractory dermatophytosis caused by locally isolated multidrug-resistant *T. indotineae* in China, resistant to terbinafine, fluconazole, and griseofulvin, with some strains also exhibiting cross-resistance to itraconazole and posaconazole (9). This rapid emergence of multi-resistant human pathogens further complicates treatment and limits therapeutic options, suggesting that there is an urgent need to develop highly efficient therapeutic strategies for *T. indotineae* infection.

Topical treatment may help to improve the lesions, but systemic treatment is necessary to potentially cure *T. indotineae* infections and avoid recurrence (10). The common treatment options for dermatophytosis include not only allylamines, triazoles, and griseofulvin but also topical agents, such as amorolfine, ciclopirox, and luliconazole (11). Other available antifungal drugs include polyenes (amphotericin B) (12) and echinocandins (caspofungin, micafungin, and anidulafungin) (13), which are used for treating invasive fungal diseases by targeting fungal cell membranes and inhibiting cell wall synthesis, respectively. In recent years, several novel triazole agents have emerged. Isavuconazole**,** active against a broad spectrum of yeasts, molds, and dimorphic fungi, was FDA-approved in 2015 for intravenous and oral formulations to treat aspergillosis and mucormycosis (14). Ravuconazole, the active metabolite of fosravuconazole, was approved in Japan in 2018 as an oral formulation for onychomycosis and has demonstrated excellent activity against dermatophytes (15). Oteseconazole, approved in the USA in 2022, is an oral azole for recurrent vulvovaginal candidiasis with the potential for treating onychomycosis and invasive infections (16). To determine whether the aforementioned antifungals could offer new therapeutic hope for refractory dermatophytosis caused by *T. indotineae*, it is imperative to evaluate the *in vitro* susceptibility of this emerging pathogen (17). Here, we collected 37 *T. indotineae* isolates and performed antifungal susceptibility testing (AFST) against 16 antifungals, aiming to discover potential effective treatments and provide a basis for establishing interpretive breakpoints for *T. indotineae*.

The 23 *T. indotineae* isolates were obtained from the Hospital of Dermatology, Chinese Academy of Medical Sciences, between February 2021 and December 2024. Additionally, 14 clinical isolates from India, collected between 2018 and 2019, were included in the study (18). All 37 isolates were identified based on morphological characteristics (19) and internal transcribed spacer (ITS) sequencing (20). We tested 16 antifungals (Merck), grouped into two categories: nine commonly used for dermatophytosis and seven other antifungals. AFST was performed using the CLSI M38-A3 broth microdilution method (21). Drug solutions were dissolved in dimethyl sulfoxide (DMSO), and serial twofold dilutions were prepared in 1× RPMI 1640 medium. Conidial suspensions (2–6 × 10³ CFU/mL) were prepared and inoculated into drug dilution plates. After 96 h of incubation at 35°C, MICs or minimum effective concentrations (MECs) were visually determined (22). Subsequently, correlations between the MICs of azoles were assessed using Spearman’s rank correlation coefficient (r), a nonparametric measure that evaluates the strength and direction of a monotonic relationship between two variables (4, 23). To determine whether there were significant differences in MICs between isolates from China and India, the Kruskal-Wallis test was performed, as MICs data were not normally distributed (24). Due to the wide variability in MIC ranges among different antifungal agents, the MICs on the *Y*-axis in the scatter plots were divided into three segments to enhance visualisation. *P*-value < 0.05 is considered statistically significant.

The MIC ranges, geometric mean (GM), MIC50, and MIC90 values for 16 antifungals against 37 isolates were determined (Table 1). Among the commonly used agents for dermatophytes, luliconazole exhibited the highest efficacy against *T. indotineae*, with the lowest GM value (0.005 mg/L), followed by amorolfine (0.160 mg/L), posaconazole (0.265 mg/L), voriconazole (0.308 mg/L), itraconazole (0.325 mg/L), ciclopirox (0.464 mg/L), griseofulvin (1.350 mg/L), terbinafine (4.756 mg/L), and fluconazole (50.166 mg/L). The MICs for terbinafine, fluconazole, and griseofulvin were significantly elevated (*P <* 0.0001 for all comparisons), consistent with clinical resistance patterns, whereas the MICs for itraconazole, posaconazole, and voriconazole only showed a modest increase, indicating that they may still be considered for systemic therapy. Among the other antifungals tested, caspofungin, micafungin, and anidulafungin exhibited markedly low MECs (*P <* 0.0001), indicating strong *in vitro* inhibition of *T. indotineae* hyphal growth. Nonetheless, this does not necessarily translate into clinical efficacy, as echinocandins are primarily fungistatic rather than fungicidal against dermatophytes, and their limited penetration into keratinized tissues further restricts their potential utility in treating dermatophyte infections (5). Additionally, *T. indotineae* isolates displayed reduced susceptibility to amphotericin B (3.575 mg/L), oteseconazole (1.276 mg/L), isavuconazole (0.982 mg/L), and ravuconazole (0.530 mg/L).

**TABLE 1:** *In vitro* susceptibilities of 37 *Trichophyton indotineae* isolates to 16 antifungal agents, determined by CLSI methodology

Classification	Drugs	MIC^a^ (mg/L)				
		Range	Mode	MIC_50_	MIC_90_	GM
Common antifungals for dermatophytes	Terbinafine	0.002–≥ 128	≥ 128	≥ 128	≥ 128	4.756
	Fluconazole	4–≥ 128	128	≥ 128	≥ 128	50.166
	Itraconazole	0.032–2	0.5	0.5	0.5	0.325
	Voriconazole	0.032–1	0.5	0.5	1	0.308
	Posaconazole	0.063–0.5	0.5	0.25	0.5	0.265
	Luliconazole	≤0.0003–0.02	0.01	0.01	0.02	0.005
	Griseofulvin	0.125–4	4	1	4	1.350
	Amorolfine	0.016–0.5	0.25	0.125	0.5	0.160
	Ciclopirox	0.25–1	0.5	0.5	0.5	0.464
Other antifungal agents	Ravuconazole	0.032–2	2	1	2	0.530
	Isavuconazole	0.063–4	2	2	4	0.982
	Oteseconazole	0.125-4	2	2	2	1.276
	Amphotericin B	2–4	4	4	4	3.575
	Caspofungin ^*b*^	≤0.002–0.063	≤0.002	≤0.002	≤0.002	≤0.002
	Micafungin ^*b*^	≤0.001–0.032	0.004	≤0.001	0.004	0.001
	Anidulafungin^*b*^	0.002–0.063	0.004	0.004	0.004	0.003

^*a*^ MIC measures the activity of antifungal agents (except echinocandins), defined as the minimum inhibitory concentration achieving 100% growth inhibition for amphotericin B and 80% inhibition for other agents.

^*b*^ Echinocandins use MEC, defined as the minimum effective concentration where small, rounded, highly branched hyphal clusters appear. Common antifungals for dermatophytes are those routinely used for treating dermatophytosis. Other antifungal agents refer to systemic drugs mainly used for invasive fungal infections or non-dermatophyte pathogens.

**Fig 1:**
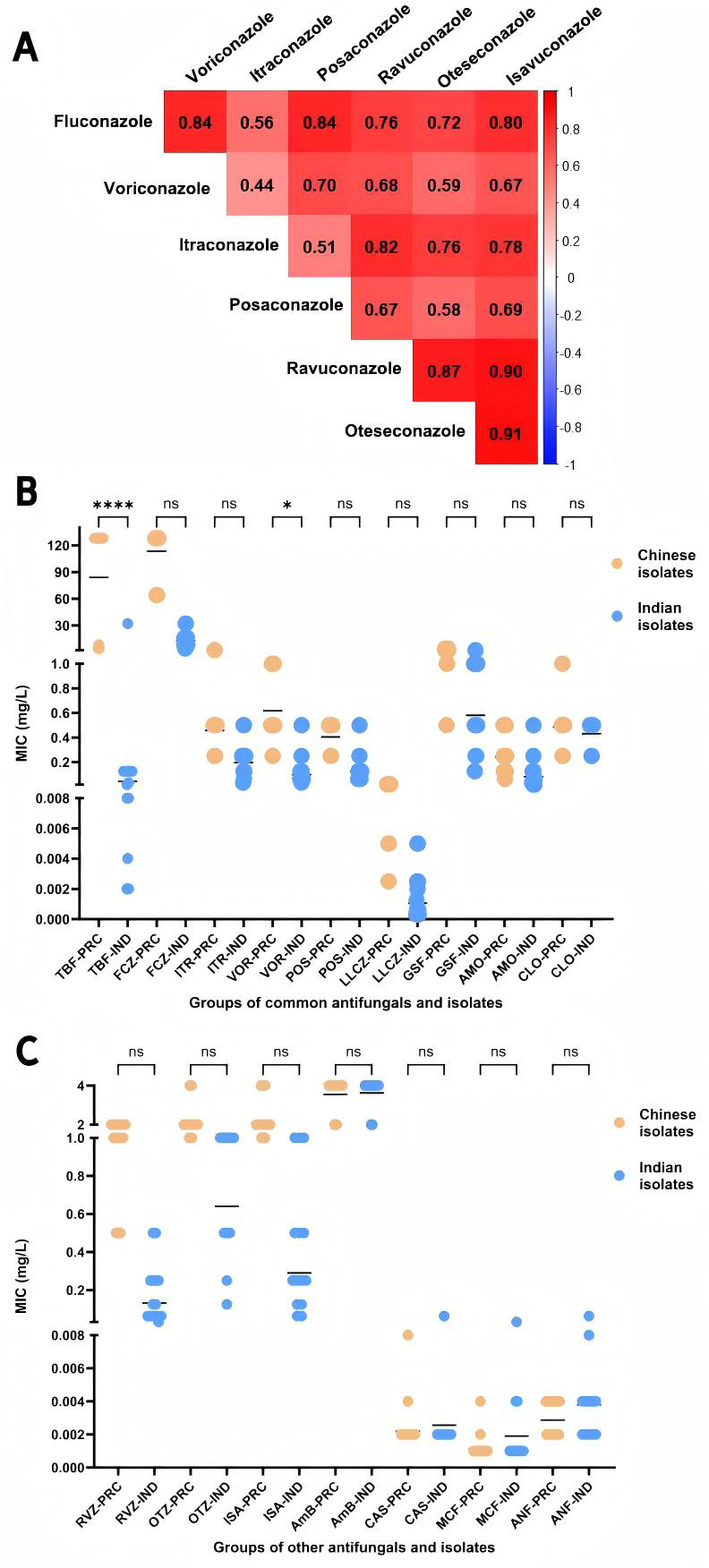
(**A**) Heatmap (R version 4.4.1) of Spearman’s rank correlation coefficients for the MICs of six azoles against *T. indotineae* isolates. Correlation coefficients range from −1 (strong negative correlation) to +1 (strong positive correlation). Color intensity indicates correlation strength, with deeper red representing stronger positive correlations. (**B**) Scatter plots (GraphPad Prism version 10.1.2) comparing the MICs of nine commonly used antifungals for dermatophytosis between Chinese and Indian *T. indotineae* isolates. Abbreviations: TBF (terbinafine), FCZ (fluconazole), ITR (itraconazole), VOR (voriconazole), POS (posaconazole), LLCZ (luliconazole), GSF (griseofulvin), AMO (amorolfine), CLC (ciclopirox), PRC (Chinese isolate), IND (Indian isolates). (**C**) Scatter plots comparing the MICs of seven other antifungals between Chinese and Indian *T. indotineae* isolates. Abbreviations: RVZ (ravuconazole), OTZ (oteseconazole), ISA (isavuconazole), AmB (amphotericin B), CAS (caspofungin), MCF (micafungin), ANF (anidulafungin), PRC (Chinese isolates), IND (Indian isolates). The *x*-axis represents the tested drugs, grouped by category, while the *y*-axis shows MIC (mg/L) plotted on a segmented, non-continuous linear scale to accommodate the wide range of data. Statistical significance was determined using the Kruskal-Wallis test (*P < 0.05, ****P < 0.0001).

Spearman correlation coefficient analysis revealed distinct patterns in the MICs of azoles, providing insights for optimizing antifungal treatment choices (Fig. 1A). Strong positive correlations were observed between the MICs of ravuconazole, oteseconazole, and isavuconazole. Fluconazole also demonstrated significant positive correlations with voriconazole, posaconazole, and isavuconazole. These highly correlated drug pairs indicate high degrees of consistency in their MIC changes, suggesting that these azoles might exhibit similar antimicrobial activity and therapeutic effects against *T. indotineae*. In contrast, the correlation between itraconazole and the other azoles, including fluconazole, voriconazole, and posaconazole, was weak. This indicates that itraconazole’s MICs do not follow the same trend, possibly reflecting differences in its antifungal activity or resistance mechanisms.

There are some differences in the antifungal susceptibility of *T. indotineae* isolates from China and India. The MIC values for terbinafine (*P* < 0.0001) and voriconazole (*P* = 0.0205) were significantly higher in the Chinese isolates compared to those from India (Fig. 1B), while both Chinese and Indian isolates exhibited similar susceptibility levels to other 14 antifungals, including itraconazole, ciclopirox, amphotericin B, and echinocandins (Fig. 1C). The higher terbinafine and voriconazole MICs in Chinese *T. indotineae* isolates might reflect combined effects of temporal and epidemiological differences and clonal transmission. Chinese strains were collected more recently, possibly after prolonged antifungal exposure. Extensive terbinafine use, alongside indirect azole pressure from voriconazole use in healthcare and agricultural azole fungicides, may have driven resistance. Additionally, our recent molecular epidemiological studies indicated that Chinese isolates likely represent secondary cases introduced from South Asia, followed by local clonal transmission which could amplify this trend (25). There is also minor bias due to the limited number of strains, highlighting the need for continued strains collection and resistance monitoring.

Our results revealed that *T. indotineae* not only exhibited resistance to commonly used antifungals but also decreased susceptibility to novel azoles and amphotericin B, with regional resistance variations. The strong correlation between certain azoles suggested potential cross-resistance, possibly limiting treatment options. Fortunately, the topical agents luliconazole and amorolfine demonstrated low MICs, and the slight increase in the oral formulation itraconazole’s MICs indicated that they could be effective for treating *T. indotineae* infections. Echinocandins demonstrated favorable *in vitro* activity against *T. indotineae*; however, given their limited clinical use and lack of established efficacy in dermatophytosis, their potential as therapeutic alternatives for resistant infections warrants further investigation. This study offers updated insights into treatment options and enhances understanding of antifungal resistance in *T. indotineae*. Nonetheless, the lack of established epidemiological cut-off values, pharmacokinetics and pharmacodynamics, and clinical breakpoints limit accurate resistance assessment and treatment guidance. Further research is essential to monitor antifungal resistance trends and optimize treatment strategies for *T. indotineae*.
